# PPARγ and Cognitive Performance

**DOI:** 10.3390/ijms20205068

**Published:** 2019-10-12

**Authors:** Michele d’Angelo, Vanessa Castelli, Mariano Catanesi, Andrea Antonosante, Reyes Dominguez-Benot, Rodolfo Ippoliti, Elisabetta Benedetti, Annamaria Cimini

**Affiliations:** 1 Department of Life, Health and Environmental Sciences, University of L’Aquila, 67100 L’Aquila, Italy; 2 Sbarro Institute for Cancer Research and Molecular Medicine and Center for Biotechnology, Temple University, Philadelphia, PA 19122, USA

**Keywords:** PPAR gamma, Parkinson’s disease, Alzheimer’s disease, autism, schizophrenia, Huntington, PPARγ agonists

## Abstract

Recent findings have led to the discovery of many signaling pathways that link nuclear receptors with human conditions, including mental decline and neurodegenerative diseases. PPARγ agonists have been indicated as neuroprotective agents, supporting synaptic plasticity and neurite outgrowth. For these reasons, many PPARγ ligands have been proposed for the improvement of cognitive performance in different pathological conditions. In this review, the research on this issue is extensively discussed.

## 1. Introduction

Peroxisome proliferator-activated receptors (PPARs) are included in the superfamily of nuclear hormone receptors (NHR). Three different PPAR isotypes (PPARα, PPARβ, also called δ, and PPARγ) have been recognized in different species and are structurally homologous. PPARs exert a key role in pathophysiological processes of several disorders of the central nervous system (CNS). In rat, PPARγ is abundantly expressed in dopaminergic cells of the basal ganglia [1,2]. PPARγ expression is elevated in some areas of the CNS, such as the cortex, olfactory tubercle, thalamic nuclei, and in the reticular formation [3]. More recently, a deep investigation on PPAR expression in mouse and human brain demonstrated that PPARγ is mainly localized to neurons and astrocytes [4], and that it is expressed in most of the brain regions, with greater expression in the prefrontal cortex, nucleus accumbens, and amygdala with respect to the ventral tegmental area.

PPARs in the CNS have been mainly associated with lipid metabolism, but PPARγ are also involved in neuroprotection, stem cells maintenance, and inflammation [5]. PPARγ in the brain controls feeding behavior; thus, it has been shown that PPARγ activation in the brain mediates some of the adverse effects of thiazolidinediones (TZD) [6,7]. Indeed, PPARγ inhibition counteracts weight gain, on the contrary to what happens upon TZD administration or a condition with PPARγ constitutively active in the hypothalamus [7]. Comparably, PPARγ ablation in neurons induces a food intake reduction and increases the energy expenditure in high-fat diet mice [6].

Recently, the focus of PPARγ in neural degenerative diseases has intensified, and many PPARγ ligands have been proposed as therapeutic approaches for the resulting cognitive impairment. However, the underlying molecular mechanisms mediated by PPARγ, counteracting cognitive decline, need to be thoroughly evaluated. Additionally, due to the limited bioavailability in the brain, the use of PPARγ ligands as drugs is still limited. Consequently, the gap in knowledge is the development of PPARγ-targeted agents characterized by higher tolerability, a goal that can be reached through understanding the molecular structure and the mechanism by which PPARγ controls various cell functions. This review is focused on the positive role of PPARγ ligands and their therapeutic potential in cognitive disorders, in particular, in different cerebral pathological conditions in which the cognitive function is impaired.

## 2. PPARγ and Cognition

Cognitive function, learning, and memory have different players, each one dependent from the other, equally crucial for cognitive performance, brain-derived neurotrophic factor (BDNF), long-term potentiation (LTP), and mitochondria. In particular, mitochondria are involved in LTP of synaptic transmission, a crucial process in learning and memory [8,9,10]. Changes in mitochondria localization and rapid functional alterations in dendrites and axons may be involved in synaptic plasticity. These variations may comprise mitochondrial Ca^2+^ movements, production of ROS (Reactive Oxygen Species), and the release of proteins and other factors.

Increasing evidence indicates that the effects of BDNF and glutamate are also linked to mitochondria activity and synaptic plasticity. Indeed, in addition to BDNF, other neurotrophic factors linked to learning and memory processes in the hippocampus are able to modulate synaptic plasticity [11]. BDNF increases synaptic plasticity, partially by increasing mitochondrial energy production due to an increase of glucose consumption in cortical neurons in culture, in response to increased energy request [12], and enhances mitochondrial respiratory coupling [13]. In agreement with this hypothesis, BDNF expression and signaling is enhanced by environmental factors, including exercise, cognitive tasks, and diet—all these phenomena increasing cellular energy consumption [14] and relating to the activity of PPARs.

The involvement of PPARγ in cognitive performance arises from indirect findings obtained on experimental models of brain pathology. For example, it has been reported in seipin knockout mice, an in vivo model of congenital generalized lipodystrophy 2 (CGL2), that spatial cognitive deficit was abrogated by PPARγ activation, leading to increase of glutamate AMPA (α-amino-3-hydroxy-5-methyl-4-isoxazolepropionic acid receptor) receptor, ERK2 (mitogen-activated protein kinase 2) phosphorylation, and phosp5ho-cAMP response element binding protein (CREB). In fact, neuronal seipin deficiency, reducing ERK (Extracellular signal-regulated kinases)-CREB (cAMP response element-binding) activities, selectively suppresses AMPAR (α-amino-3-hydroxy-5-methyl-4-isoxazolepropionic acid receptor) expression, compromising LTP and spatial memory, which may be counteracted by PPARγ activity. Impairment in learning and memory and in synaptic plasticity are closely related with aging [15], which is also linked to the release of pro-inflammatory cytokines [16], which in turn alters LTP. PPARγ activation by rosiglitazone (RSG) was shown to improve the age-dependent decrease of LTP [17]. Chronic RSG administration rescued LTP impairment in 22-month-old rats at the synapses of the perforant path-dentate gyrus (PP-DG), indicating that anti-inflammatory drugs could rescue synaptic plasticity [18]. Other authors described how RSG improved neurocognitive deficits depending on aging in older animals [19]. They showed that, at the behavioral level, acute and chronic RSG administration increased learning ability. In parallel, synaptic plasticity in the dentate gyrus of RSG-treated rats was restored. Also, the hippocampal area devoted to spatial receptive fields was ameliorated by chronic RSG administration. In addition, in aged rats, RSG administration improved hippocampal glucose transporter (GLUT)-3 expression. Altogether, these data indicated that RSG affected hippocampal activity, inducing an improvement of cognitive performance, likely by the up-regulation of glucose intake by GLUT-3.

In the chronic cerebral hypoperfusion (CCH) model, it has been shown that PPARγ activation by Icariside II, a plant polyphenol, enhanced the expression of BDNF, tyrosine receptor kinase B (TrkB), Akt, and cAMP response element binding protein (CREB) phosphorylation, leading elements of LTP and synaptic plasticity [20].

This phenomenon has also been reported in the rare disorder Fragile X syndrome (FXS), a disease leading to intellectual disability and altered synaptic plasticity. Recent studies have revealed a direct link of nuclear receptors with mental retardation and neurodegenerative diseases. In particular, PPARγ agonists, by promoting synaptic plasticity and neurite outgrowth, have been identified as good candidates for therapeutic intervention in intellectual disabilities. Preliminary data obtained indicated that pioglitazone (PGL), RSG, and synthetic agonist GW1929, are involved in intracellular transduction signals (e.g., Phosphatidylinositol-3-Kinase/ protein B kinase (PI3K/Akt), GSK3b (Glycogen Synthase kinase 3 beta), MMP-9 (Matrix Metalloproteinases 9), Rac1(Ras-related C3 botulinum toxin substrate 1) and Wnt (Wingless-related integration site)/β-Catenin). In FXS animal models [21], these pathways are linked to memory recognition and enhanced synaptic plasticity, suggesting an improvement in learning and memory processes in this syndrome.

### PPARγ and BDNF

BDNF plays a fundamental role for learning and memory and is generally downregulated or not correctly truncated in several neurological diseases characterized by cognitive impairment. Recent findings have reported the involvement of activated PPARγ in modulating BDNF levels in different pathologies [22,23]. Immunocytochemistry studies from Amin and collaborators confirmed that the activation of PPARγ induced an increase in BDNF expression [24,25]. To validate whether this observation is a direct consequence of PPARγ activity, the authors transiently transfected a constitutively active PPARγ (CA-PPARγ) vector into H19-7 cells and revealed increased immunodetection of BDNF in H19-7 cells [24,25]. Finally, cognitive impairment may result also from hypertension, and is dependent on the function of the hippocampus [26]. BDNF protects against cell death in the hippocampus. It has been reported that angiotensin II receptor blockers (ARB) increase BDNF in the hippocampus. Telmisartan, a unique ARB with PPARγ-stimulating activity, has been shown to counteract cognitive impairment through the up-regulation of BDNF/TrkB in the hippocampus, partially due to its ability to activate PPARγ [25]. Moreover, type 2 diabetic (db/db) mice treated with RSG through the pharmacologic activation of hippocampal PPARγ, showed a significant improvement in memory, long-term potentiation, and post-tetanic potentiation, but did not ameliorate peripheral insulin sensitivity. Central PPARγ stimulation induced the expression of BDNF and thus suggested a potential molecular signaling pathway, improving cognitive deficits associated with diabetes related to PPARγ [25].

Future work will investigate the significance of the PPARγ-BDNF signaling on ameliorating behavior and synaptic plasticity in neurological diseases by inhibiting the BDNF receptor (TrkB).

**Figure 1:**
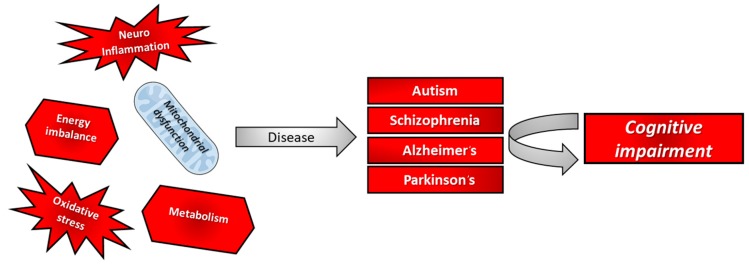
Cognitive impairment causes in some neurological diseases.

The majority of the data available linking PPARγ with cognitive performance derives from studies on neurological diseases (Figure 1), where once again the role of PPARγ activation in cognition is apparent.

## 3. PPARγ and Alzheimer’s Disease

Alzheimer’s disease (AD) patients are significantly increasing in recent years, presenting a substantial challenge to healthcare systems. AD hallmarks are represented by the formation of intracellular neurofibrillary aggregates of tau protein and extracellular plaques of amyloid-β peptides, leading to neuronal death, responsible for the cognitive function decline and progressive memory impairment [27,28]. Under physiological conditions, PPARγ expression in the brain is relatively low. Nevertheless, PPARγ levels are higher in AD individuals, indicating that PPARγ performs a crucial role in regulating AD pathology [29,30]. However, data on active PPARγ in the pathology are not conclusive; in fact, these authors described the increase of PPARγ mRNA, but did not demonstrate its activation, nor its nuclear localization. It is possible to speculate in this case that the observed increase of PPARγ mRNA may be due to an adaptive mechanism aimed to counteract Aβ increase, due also to the known role of PPARγ in controlling insulin degrading enzyme (IDE) expression. However, several studies have reported that PPARγ agonists produce positive effects in AD in patients and in in vivo models as well, thus supporting a protective role of the active transcription factor in this disease [31].

Cognitive impairment is one of the main features of AD patients and AD animal models. It has previously been shown that therapeutic approaches using PPARγ agonists ameliorated cognition and memory in transgenic AD animal models [32,33]. In addition, meta-analysis studies reported that PGL may counteract the cognitive impairment in patients with early or mild-to-moderate AD [34]. Other findings provide support that RSG is able to counteract AD-associated cognitive decline Indeed, RSG ameliorates hippocampal cognitive deficits in some AD patients, and counteracts cognitive deficits in the Tg2576 mouse model for AD amyloidosis through PPARγ signaling activation and ERK cascade, a key mediator for memory consolidation [35].

Cognitive impairment in AD is linked to neuroinflammation, increased Aβ levels, functional network connectivity, oxidative stress, and mitochondrial dysfunctions [28,36,37]; thus, potential therapeutic approaches should restore these pathways, counteracting or ameliorating AD-associated cognitive dysfunctions. PPARγ plays a key role in most of the pathways involved in AD and, indeed, PPARγ agonists represents a potential therapeutic approach able to restore or improve cognitive performances. 

PPARγ agonists are also able to reduce Aβ levels, increasing its clearance or decreasing the Aβ formation, as reported by several studies. For instance, APP (amyloid precursor protein)-transgenic mice upon PGL treatment showed lower expression of β-secretase, a protease POLI that cleaves APP to generate Aβ [38]. Additional studies reported that APP processing and Aβ production are not altered by PGL, indicating that PPARγ agonists reduce Aβ levels through Aβ clearance or increasing apolipoprotein E (ApoE) concentrations in the brain [39]. 

In the APP/presenilin-1 mouse model, DSP-8658, a selective PPARα/γ modulator, and PGL were tested. Both treatments activate PPARγ, leading to improved microglial Aβ phagocytosis, mediated by scavenger receptor CD36, which induces lower cortical and hippocampal Aβ levels. Moreover, DSP-8658 treatment improves spatial memory performance [40]. Consequently, stimulation of microglial clearance by concomitant activation of the PPARγ/RXRα (Retinoid X receptor alpha) heterodimer has been indicated as a potential approach in AD prevention, leading to improved cognitive performances.

Another hallmark of AD is chronic neuroinflammation without leukocyte infiltration [30]. The concomitant activation of microglia and the inflammatory molecule release can impact neuronal loss and stimulate AD progression [41,42]. Besides microglia activation, glial- and astrocyte-derived inflammatory molecules and inflammatory enzymes such as iNOS (inducible nitric oxide synthase) may also contribute [43,44,45]. 

For instance, the first studies on PPARγ and AD linkage discovered the ability of non-steroidal anti-inflammatory drugs (NSAIDs) in dampening the neuroinflammation and activating PPARγ, thereby reducing AD risk by more than 80% [43,46,47,48,49]. Other investigations in vitro and in vivo confirmed this hypothesis and suggested that NSAIDs provide a therapeutic effect by binding to PPARγ present in the AD brain, thus inhibiting iNOS expression and neuronal cell death [5,50]. SNU-BP, an agonist of PPARγ, inhibits lipopolysaccharide (LPS)-induced NO (Nitrogen Oxide) production and pro-inflammatory cytokine production by PPARγ activation [51]. SNU-BP potentiates interleukin 4-induced arginase-1 expression, promoting microglial polarization toward an M2 anti-inflammatory phenotype [51]. 

It is also necessary to take into account that the anti-inflammatory effects of PPARγ agonists may be independent of its activity. Indeed, in some cases, PPARγ expression does not correlate with the anti-inflammatory activities observed. On the other hand, in PPARγ, KO modeling the anti-inflammatory activity of TZDs was not observed, whereas inflammatory cytokines were also inhibited equally in PPARγ-deficient macrophages [52]. These results indicated that some anti-inflammatory effects of TZDs are independent of PPARγ activity and that there is a significant gap in knowledge regarding the mechanism by which TZDs and PPAR exert anti-inflammatory activities in AD. In any case, these studies indicated that PPARγ might be an essential target for dampening neuroinflammation in AD through the expression of proinflammatory genes [53,54]. Another feature of AD pathogenesis is neuronal death and altered energetic metabolism caused by mitochondrial degradation and dysregulated trafficking. Indeed, Aβ accumulated in the mitochondrial membrane may trigger the intrinsic apoptotic pathway, inducing neuronal death [52,55]. PPARγ coactivator 1 alpha (PGC1-α) is expressed at a high level in the brain and represents a key regulator of energy metabolism and mitochondrial biogenesis; in AD brain, PGC1-α expression is significantly decreased [56]. Reduced levels of PGC1-α trigger a decreased mitochondrial density in different brain regions, including the cortex, cerebellum, and midbrain, concomitantly with decreased ATP levels [57]. In vitro studies indicated that RSG activates PGC1-α signaling pathway, thus increasing mitochondrial function and mass, and counteracting cognitive dysfunction [58]. 

In an AD animal model, PGL acts in activating antioxidant, anti-inflammatory, anti-apoptotic, and neurogenesis-like pathways, thus reverting memory loss and oxidative stress and restoring BDNF levels [59]. These investigations indicated that one of the possible mechanisms by which PPARγ ligands improve mitochondrial functions and cognitive performance is the increase of neurotrophin supply.

Another critical AD feature involves insulin signaling, as is essential to ensure neuronal survival and homeostasis, therein supporting learning and the memory formation process [60,61,62,63,64]. Interestingly, post-mortem studies in AD individuals reported low levels of insulin and insulin-like growth factors (IGF), as well as its substrate (IRS-1) in the brain [65]. A dysregulation in the insulin signaling triggers to impair neuronal oxidative metabolism [66]. In high-fat diet (HFD) rats, RSG treatment reverts neuronal insulin resistance and decreases mitochondrial ROS production, mitochondrial membrane potential variations, and mitochondrial swelling in the brain. These findings suggest that HFD-induced neuronal insulin resistance and brain mitochondria dysfunctions can be reversed by RSG [30,67], improving cognitive functions. One of the main AD genetic risk factors, apo E4, plays a pivotal role in multiple neuropathological pathways, involving dendritic spine structure and mitochondrial function alteration, leading to cognitive impairment. In rat cortical neurons, RSG significantly reduced dendritic spine density in a dose-dependent way. This event was reverted by GW9662, a PPAR specific antagonist, indicating that it was PPARγ-dependent. 

In addition, PGL decreases N-methyl-d-aspartate (a glutamate agonist)-mediated calcium currents and transients, characteristic of AD models, improving cognitive performances, tested through Y-maze test and passive avoidance tests, in scopolamine-induced memory impairment mice [68]. PPARs could positively influence AD pathology. In fact, in AD brain, PPARγ agonists can directly improve the processing of the Aβ peptide by regulating insulin degrading enzyme (IDE), but can also regulate the inflammatory status and the mitochondrial function by modulating oxidative stress. 

Nonpharmacological strategies for the prevention of AD are associated with lifestyle interventions, including exercise, mental challenges, and socialization, as well as caloric restriction and a healthy diet [69]. Indeed, physical activity was associated with the reduction of AD deficits in a cohort study [70]. 

Lifestyle intervention was reported to enhance hippocampal neurogenesis and learning in aging rodents. The mechanisms proposed in order to explain the neuroprotective effect of lifestyle are (A) the release of neurotrophic factors (such as BDNF and IGF-1) from neurons during synaptic activity, which stimulate neurogenesis and synaptic plasticity; (B) the reduction of free radicals in the hippocampus; and (C) peripheral signals that support the demands of active neuronal networks such as BDNF release [71].

The results collected so far from in vitro and in vivo models highlight the positive effects of PPARγ agonists in ameliorating cognitive performances in AD, and propose these drugs, or natural ligands of PPARγ, as future therapeutic approaches in cognitive impairment. 

## 4. PPARγ in Parkinson’s Disease

Parkinson’s disease (PD) is a progressive, chronic, and debilitating disorder and represents the second most common neurodegenerative disease after AD. The main histological hallmarks are the degeneration of dopaminergic neurons in the *substantia nigra* and the presence of fibrillar aggregates, named Lewy Bodies. PD is characterized by motor dysfunctions (including bradykinesia, rigidity, and impaired imbalance) and non-motor dysfunctions (cognitive impairment, gastrointestinal abnormalities, and depression) [72,73]. 

PPARγ is involved in PD symptoms, as reported in an interesting paper, in which experimental hemi-PD rats (6-OHDA (6-hydroxydopamine)) showed a strong downregulation of PPARγ level in the lesioned brain areas [74].

Cognitive dysfunction is one of the most frequent non-motor symptoms in PD patients [75], around 20%–33% suffer mild cognitive impairment at PD diagnosis [76,77], and more than 60% show Parkinson’s disease dementia within 12 years of disease extent [78]. 

Neuroinflammation and activated microglia are involved in the onset of cognitive decline in PD patients [79]. Indeed, in the substantia nigra of PD patients, elevated levels of reactive microglia have been reported [80,81,82]. However, it is still unclear if the neuroinflammation is the trigger or a consequence of PD. Anti-inflammatory approaches may have potential in counteracting PD progression [83]. On this line, PPARγ agonist has been successful in neurodegenerative disease, dampening the inflammation typical of PD patients. It has been reported that NSAIDs are able to prevent or counteract PD, dampening of the neuroinflammation in different PD models, reducing the risk [80,84]. On the other hand, there are controversial results from other studies, indicating that NSAIDs are not able to reduce PD risk [84,85,86,87]. 

Concerning PPARγ agonist treatments such as PGL, positive results in PD model have been reported [88]. PGL counteracted dopaminergic loss and neuroinflammation, but was not able to reduce microglial activation or restore dopamine levels [89]. In an in vitro study, PGL exerted a neuroprotective effect, reducing NO production, iNOS expression, cyclooxygenase-2 (COX-2), and p38 MAPK (Mitogen-Activated Protein Kinase) activity [90,91]. Natural and synthetic PPARγ agonists, in LPS-stimulated microglial cultures, were able to decrease inflammation and neurotoxicity, reducing different IL (Interleukins), TNF-α (Tumor Necrosis Factor), iNOS, and IFNγ (Interferon gamma)-induced expression of major histocompatibility complex (MHC) class II antigen [92,93,94,95,96,97]. In another study, aged RSG-treated rats showed a reduction in impaired long-term potentiation, concomitant with attenuation in the LPS-induced increase in MHC-II and IL-1β in cells extracted from wild-type mice, while this effect was not observed in culture IL-4^−/−^ mice-derived [17]. Noteworthy, in a chronic MPTP (1-methyl-4-phenyl-1,2,3,6-tetrahydropyridine) model of PD, PGL through PPARγ, induced a reduction in inflammatory and antioxidant pathways, reducing dopaminergic cell loss [88,98]. Notably, PGL inhibited NFkB (Nuclear Factor kinase B), iNOS, and NO. The involvement of NFkB signaling was reported to be involved, not only in the neuroinflammatory response, but also in oxidative stress [99,100].

In MPTP mouse model, the neuroprotection reported upon PGL pretreatment was due to the block in the conversion of MPTP to its active toxic metabolite MPP+ (1-methyl-4-phenylpyridinium) by the inhibition of the enzyme monoamine oxidase B (MAO-B) [101]. Moreover, on the same PD model, other studies reported that PGL prevents nitrotyrosine storage and glial activation [88,89,102]. 

On the other hand, in another PD model obtained by intra-striatal lipopolysaccharide (LPS) infusion, in addition to the glial inhibition, the positive effect of PGL was due to the decrease of mitochondrial protein uncoupling protein 2 (UPS2) and the mitochondrial protein mitoNEET [103].

Concerning RSG treatment, in a mouse model of progressive PD, RSG was able to completely inhibit microglia activation in the substantia nigra, concomitant with complete preservation of DA (Dopamine) cell bodies, but only partially in the striatum, with only partial rescue of DA content decrease [104]. Interestingly, contrary to what was observed by Quinn and collaborators, in the same investigation, the authors reported that RSG did not alter MPP+ levels, indicating that RSG-induced neuroprotective effects were not mediated by blockade of MAO-B activity [104].

In the substantia nigra of PD patients, higher levels of P53, TNFα, and active caspases were found, suggesting the involvement of apoptosis, in addition to neuroinflammation and activation of neuroglia [105].

In line with these results, MPTP models showed active caspase-3, and neuroprotective therapeutic approaches can prevent caspase-3 activation and neuronal apoptosis [106]. 

In in vitro and in vivo models, PGL treatment inhibits caspase-3 activation [89] and promotes PGC-1*α*, a transcriptional coactivator of PPARγ, restoring mitochondria ultrastructure and reducing dopaminergic neuron damage [89,107]. These findings indicate that PGC-1α also contributes to the neuroprotection of dopaminergic neurons and may further elucidate the neuroprotective role of PGC-1α (a mitochondrial biogenesis regulator), leading to a novel therapeutic approach for PD. 

PD is also characterized by oxidative stress and impaired mitochondrial function [108,109,110,111]. RSG was able to protect against mitochondrial alteration MPP(+)-induced, thanks to its anti-oxidative and anti-apoptotic activities, notably enhancing SOD and catalase, as well Bcl-2 (B-cell lymphoma 2) and Bax (BCL2-associated X protein) expression [112]. Another study confirmed the mitochondria as major targets of PPARγ, and that PGL maintains the mitochondrial function. In particular, the authors reported that PGL enhanced PGC-1α expression and UCP2 (Uncoupling Protein 2) (a mitochondrial protein that reduces ROS production) level, as well as COX-1, and prevented TNFα effects [113]. 

In two in vitro models, differentiated SH-SY5Y cells with chronic partial inhibition of complex I through rotenone and PINK1 (PTEN-induced kinase 1 gene) knockdown cells, pretreatment with RSG restores mitochondrial biogenesis, prevents free radical generation and autophagy, and increases oxygen consumption [114]. A novel thiobarbituric-like compound MDG548, a functional PPARγ agonist, counteracts H_2_O_2_ and MPP^+^ neurotoxicity in MPTP in vitro and in vivo models. Moreover, MDG548 decreased the reactive microglia and iNOS induction in the substantia nigra of the animals [115]. Overall, as MDG548 is not a TZD compound but presents high PPARγ affinity, it represents a safe alternative agonist for PD.

Another interesting investigation reported that MPTP rats showed poor cognitive performance in the Morris water maze test and passive avoidance task, linked to a strong increase in oxidative stress. Chronic administration of PGL was able to improve cognitive performance in both behavioral tests, also reducing oxidative stress [116]. 

Lifestyle habits were reported to ameliorate and counteract PD progression [117]. Indeed, a recent study reported that omega-3 and vitamin E through the up-regulation of PPARγ improved some clinical symptoms of PD [118]. Moreover, evidence suggests that regular physical exercise programs may exhibit potential benefits over PD, via BDNF and PPARγ expression. Furthermore, dietary polyphenols, in particular flavonoids, may exert beneficial effects on the central nervous system by downregulating ROS levels, thus representing a potential tool to preserve cognitive performance through senescence [119]. Most of the investigation concerning the ability of PPARγ agonists to counteract neurodegeneration has been widely performed in PD models. Future experimental, preclinical, and clinical studies are necessary to deepen their efficacy on PD progression. Moreover, as described above, numerous molecular pathways are involved in PD pathophysiology and thus may explain why the current therapies are not able to fully contain PD progression because they cannot modulate all the involved pathways. The combination of different therapies with synergic effects may help in overcoming this issue. 

## 5. PPARγ in Huntington Disease

Huntington disease (HD) is a progressive brain disorder, characterized by loss of cognitive performance, uncontrolled movements, and neuropsychiatric disturbances. HD is an autosomal dominant disease, and its main cause of is a CAG (Cytosine Adenine Guanosine) trinucleotide expansion in the Huntingtin (Htt) gene. This mutation in the Htt gene causes an accumulation of mutated protein aggregates in the brain, causing neuronal degeneration [120]. HD features are represented by a decline in cognitive function, associated with a progressive loss of motor control due to a gradual degeneration of the spiny neurons of the striatum and of the pyramidal neurons present in the cortex. Depression, irritability, suicidality, apathy, anxiety, and perseverative behavior are some of the most frequently reported behavioral alterations in HD. Several studies [121,122] have shown that psychiatric manifestations and brain changes may precede the first motor signs characteristic of the disease by several years. In patients and animal models of HD, emotional functions and rules have not been well-characterized and, in fact, contrasting effects are often reported. Indeed, the first signs and pathological changes in HD are always located in the portions of the striatum [121,122]; initially, the cognitive and psychiatric aspects are affected rather than the motor aspects.

Several forms of evidence indicate that the inhibitory and excitatory processes could be altered by excessive activity within the glutamate and dopamine pathways in the basal ganglia [123], thus contributing to the onset of HD symptoms. This imbalance of neurotransmitters could represent the major cause of cell death through an excitotoxic mechanism; in fact, excesses of dopamine production have been identified in the *substantia nigra-pars compacta* in HD [123]. The benefits of PPARγ agonists have been investigated in numerous HD models. Remarkably interesting are the studies on RSG as potential approaches to treat HD patients. In fact, different researchers have indicated that RSG activity has a crucial role in metabolism and neuroprotection. Chiang et al. [124] showed that PPARγ is involved in energy homeostasis and has been identified as a possible neuroprotective target for this pathology. The protective potential of RSG was tested on the murine model R6/2 of HD to verify the possible protective activity on weight loss, motor deterioration, and formation of huntingtin aggregates. In this HD model, RSG is able to induce the expression of B-cell lymphoma 2 (Bcl-2) and BDNF, as well as inhibiting huntingtin aggregates, and restoring to normal levels the expression of PGC1α, nuclear respiratory factor 1-2 (NRF1-2), and mitochondrial transcription factor A (TFAM) in the transgenic mouse R6/2 cortex. In R6/2 mice, RSG treatment ameliorated the motor behaviors, demonstrated by three different behavioral tests (rotarod, hind-limb clasping phenotype, and gait analysis).

Another study by Jin [125] and collaborators showed that PPARγ and PGC-1 mRNA level were diminished in the striatum and cerebral cortex of HD N171-82Q mice. Furthermore, the protective effect of RSG on cells with mutant HTT was mediated by activation of the PPARγ. These results indicated that it is possible to restore the deficiency of BDNF, PGC-1, and Sirt6 (Sirtuin 6) in a HD mouse model through the chronic administration of RSG. These changes lead to motor improvement and maintenance of glucose homeostasis. Sirt6, belonging to the sirtuin family, exerts a pivotal role in regulating metabolism and longevity. Sirt6 levels were significantly low in both cells with mutant HTT and in HD mice [125]. Sirt6 levels were restored in mutant HTT-expressing cells after treatment with RSG, suggesting that RSG is implicated in the regulation of Sirt6, thus restoring the metabolic alterations found in mice with HD. Sirt6 levels were then compared in HD mouse brains and in mutated HTT expressing cells. According to these results, it is possible to conclude that the administration of RSG can compensate for the reduction of BDNF related to HD and keep the levels of PGC-1 and Sirt6 stable in the HD mouse brain. These variations upon RSG treatment can lead to improvement in motor and glucose homeostasis. There was also a maintenance of body weight and survival in N171-82Q mice that are normally subject to these deficits [125]. On the basis of the positive effects exerted by RSG, in developing HD therapeutic approaches, targeting PPARγ pathways need to be taken into account. N171-82Q HD mice RSG-treated showed better motor abilities through BDNF, indicating that PPARγ is able to preserve this neurotrophin, maintaining neuronal functions and ameliorating cognitive and motor abilities in these mice. Mishra et al. [126] have tried to improve the beneficial effects of RSG by combining it with valproic acid (VPA). VPA, a short-chain fatty acid, is commonly used to treat convulsions with a mechanism, yet this is still not clarified; however, several authors hypothesize that VPA exerts various neuroprotective effects, including the inhibition of glutamate-induced excitotoxicity, the decrease of glycogen synthase kinase-3b (GSK-3b), and the obstruction of sodium and calcium channels, increasing GABA (gamma-aminobutyric acid) and lowering aspartate levels in the brain, the maintenance of mitochondrial function, the block of apoptosis, and the promotion of neuronal vitality. Aside from this, VPA has been shown to be a potent inhibitor of histone acetyl transferases and histone deacetylase (HDAC) activity. HDAC activity is involved in the development of several chronic neurodegenerative diseases including HD [127]. VPA inhibits the activity of HDAC by directly binding to its catalytic center [128]. Mishra et al. used quinolinic acid (QA) [129] for inducing symptoms clinically observed in HD patients on model a with male Wistar rats. This treatment was shown to mimic HD-like symptoms such as hyperactivity [130], learning deficits [131], and loss of neurons when injected into the striatum of rats. Jin et al. [126] indicated that, in N171-82Q HD mice, RSG chronic administration strongly ameliorated motor abilities, suggesting that RSG is able to counteract motor abnormality typical of HD [125]. On the same line, treatment with RSG, VPA, and their combination increased BDNF levels in QA-treated animals, relating to the benefits on animal memory abilities and motor functions. These results were further supported by numerous reports suggesting that higher levels of BDNF are linked to cognitive and motor functions in neurodegenerative diseases [132]. In this investigation, memory decline upon QA treatment, related to the increase in hippocampus AChE (Acetylcholinesterase) levels, were improved upon RSG and VPA, indicating a significant increase in memory abilities in QA-treated animals. Lower doses of QA demonstrated improved memory abilities but were not able to demonstrate a significant effect on AChE levels, which may be ascribed to their positive effects on neuroinflammation, oxidative stress, apoptosis, and BDNF levels. These findings were further supported by previous studies showing an improvement in memory performance following the administration of VPA and RSG under different neurological conditions possibly through HDAC and PPARγ pathways, respectively [33]. 

Lifestyle intervention was reported to ameliorate HD. Indeed, a recent review reported different studies underlying the positive effect exerted by antioxidants in ameliorating neuronal dysfunction and plasticity in HD [133]. Overall, the neuroprotection upon VPA and RSG administration could be related to the interaction between HDAC inhibition and PPARγ activation in QA-mediated oxidative stress, mitochondrial alterations, apoptosis, and neuroinflammation pathways. However, RSG-VPA administration led to an improvement in QA-treated animals’ memory, indicating a synergistic effect.

## 6. PPARγ and Autism

Autism spectrum disorder (ASD) is a neurodevelopmental disease involving impaired social-communication and repetitive behavior [134]. In the brain, ASD is characterized by depletion of brain GSH (Glutathione), oxidative stress, and inflammation paralleled by neuroanatomical defects. The observations in humans have also been confirmed in animal models of disease, such as valproic-treated rats or propionic-treated rats [135,136]. 

In both animal models, the beneficial effects of PPARγ agonists have been demonstrated in decreased oxidative stress, neuroinflammation, pro-inflammatory cytokines, and increased brain GSH. These changes in the biochemical and immunological parameters were accompanied by an improvement of social behavior, but also in the cognitive tests.

It is presently accepted that defects in mitochondrial functionality, particularly mt-FAO (mitochondrial Fatty Acid beta-Oxidation), are present in ASD, and that TZDs have been administered in a series of inflammatory diseases such as atherosclerosis, psoriasis, and inflammatory bowel disease [137]. PGL was able to decrease inflammatory glia response and increased glucose utilization in glial cells [138]. PGL was used in ASD due to the link existing between ASD and immune dysregulation [139] and neuroinflammation [140]. The close relationship existing between neuroinflammation and ASD have been extensively reviewed by Gladiz et al. [141]. 

On the basis of its neuroprotective and anti-inflammatory activities, in 2007, PGL was used for the first time in a clinical trial including children and adolescents ranging from 3 to 17 years.In these ASD patients, PGL treatment was effective in improving social behavior, particularly in younger patients. No adverse effects were recorded in the study period [142]. Later on, PGL was used as adjuvant treatment with risperidone in ASD patients. A 10 week randomized, double-blind, placebo-controlled trial showed that PGL was able to control behavioral symptoms of ASD [143]. The effects of PGL in ASD were mainly due to the different effects of activated PPARγ, such as reduction of brain inflammatory response and increase of mitochondrial function [144]. Although safety effects of TZD were reported in patients with type 1 diabetes [145], further investigations are needed to clarify the effects on behavior and long-term safety of TZD in ASD patients. Nonpharmacological strategies could also help to ameliorate ASD. In fact, a recent review correlates ASD and impaired mitochondrial fatty acid oxidation (FAO) with the potential roles of resveratrol in ameliorating ASD dysfunctions by PPAR activation [146].

## 7. PPARγ in Schizophrenia

Schizophrenia [147] is a psychiatric disorder characterized by various symptoms that is categorized into three distinct groups: positive (hallucinations and delusions), negative (social withdrawal, anhedonia flattened affect, lack of speech, and apathy), and cognitive symptoms (decline in cognition, and deficits in reasoning and attention). Schizophrenia, in addition to being highly debilitating, reduces the average life expectancy of a person. The biological basis of depressive symptoms are still unclear, but impaired serotoninergic and dopaminergic neurotransmissions are implicated in the pathogenesis of depression and schizophrenia [148]. 

A main factor considered to be involved in the pathogenesis of schizophrenia is BDNF. There is much evidence in literature describing a significant reduction in peripheral BDNF [149,150] concentrations in patients with schizophrenia. Very recently, Martis et al. [151] have shown that mice with BDNF deficiency exhibit a depressive phenotype. Previously, Wysokinsky et al. [152] observed a peripheral reduction in BDNF level in patients with schizophrenia with depressive symptoms. 

Another protein that appears to have a connection with the symptoms of schizophrenia is SIRT1 (Sirtuin 1), a nicotinamide-dependent deacetylase (NAD+) widely involved in glucose homeostasis, DNA repair, apoptosis, inflammation, and differentiation [153]. Studies on animal models have shown a link between reduced SIRT1 activity and depressive-like behaviors [154]. In fact, Hurley et al. [155] have already shown that the depressive behavior in rat model could be improved through a SIRT1 activator. Although the link between SIRT1 and depressive symptoms in animal models has been demonstrated in the studies of Kishi [26] and Chen [156], there is no research linking cognitive deficits and levels of SIRT1 on patients with schizophrenia. On the other hand, the association between SIRT1 and BDNF has been reported by numerous studies. In a preclinical study, it has been demonstrated that SIRT1 could regulate the combination of exon BDNF with Methyl-CpG binding protein 2, increasing BDNF transcription [157]. Indeed, SIRT1 overexpression has shown significant neuroprotective effects, increasing the level of BDNF [158]. Also, Shen et al. (2019) [159] and Chen et al. (2019) [160] showed that BDNF and SIRT1-related pathway activation produced strong effects, similar to those obtained with classic antidepressants. According to various studies showing the ability of PPARγ agonists to induce the expression of BDNF [124], it is possible to propose a possible effect of drugs, such as RSG, in improving cognitive symptoms typical of schizophrenia. In contrast to these results, there is a pilot study of Yi et al. [161] in which a group of patients with schizophrenia was treated with rosiglitazone and clozapine. In this study, 8 weeks of RSG administration had no significant benefits on cognition in schizophrenic patients. These results differ from those obtained previously by Watson et al. (2005) [162]; in fact, the authors demonstrated that in AD patients, RSG treatment for 6 months improved cognitive abilities. The contrasting results could be due to differences in study populations, RSG dosing, and treatment duration, as well as a difference in the disease [161].

On the other hand, PGL showed a positive effect. Smith et al. [163] demonstrated that PGL has high efficiency as treatment for glucose and lipid abnormalities in schizophrenic patients, reducing their depressive behavior. The results on United States patients treated with PGL showed significantly reduced depressive symptoms, as measured by the PANSS (Positive and Negative Syndrome Scale) [163]. Taking into account these findings indicating that diabetes is prevalent in schizophrenia [164], Schoepf et al. assessed the protective effects of PGL in improving schizophrenia symptoms. The case report on the effects of PGL demonstrate that this reduction is evident in overweight or metabolically impaired patients with major depressive disorder [165]. In fact, from the data obtained by Iranpour et al., PGL administration concomitant with risperidone was beneficial for patients with chronic schizophrenia, notably in decreasing negative symptoms. In clinical studies regarding PGL-treated patients, it has been shown that PANSS negative and total subscale scores improved when compared with placebo. Other than negative symptoms, positive symptoms, including depressive and psychopathological symptoms, could also be affected. Hence, to deeply understand PGL effects, all the variables of the single individual should be eliminated. 

Regarding this disease, few data are available correlating lifestyle with schizophrenia. It has been described that a diet rich in lipid compound (in particular arachidonic acid) may improve some of the symptoms developed by individuals affected by psychiatric diseases [166].

## 8. Discussion and Conclusions

All living organisms have complicated homeostatic pathways for adaption and survival to stress and disease. One of these pathways is mediated by lipid mediators resulting from cyclo-oxygenase activity in response to physiological/pathological stimuli. This may be considered as an endogenous response to the inflammation in neurodegeneration. PPARγ and its ligands have a key role in cerebral physiology and can represent promising targets for the therapy of different disorders associated with neurodegeneration and cognitive impairment. For this reason, PPARγ anti-inflammatory activity has obtained a great level of consideration, as its activation exerts a wide series of beneficial effects in different in vivo models of neurological conditions (i.e., AD, PD, HD, schizophrenia). The same activities have also been reported in psychiatric disorder models—studies from Garcıa-Bueno et al. have demonstrated the increase of 15d-PGJ2 and of PPARγ under stress conditions in the cerebral cortex [167]. The authors also demonstrated that PPARγ agonists prevented inflammation and oxidative/nitrosative stress in the cortex in these animal models [167]. 

All the evidence reported in this review point toward PPARγ as a mediator of neuroprotection, ameliorating neuroinflammation, mitochondrial function, and neurotrophin levels, thus constituting a putative treatment for cognitive decline associated with neurological diseases. It is, in fact, previously reported that RSG improves cognitive function by enhancing dendritic spine density in specific brain regions [168], probably increasing mitochondrial biogenesis and function, thus improving synaptogenesis and memory formation [168]. This is of particular importance, as in patients with schizophrenia, bipolar disorder, and major depressive disorder, mitochondrial and oxidative phosphorylation alterations have been reported [169,170].

**Figure 2:**
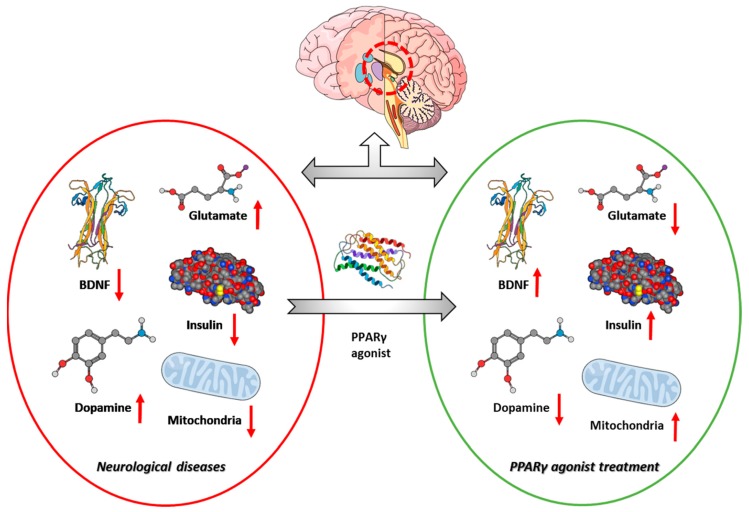
Roles of activated peroxisome proliferator-activated receptor γ (PPARγ) in the rescue of cognitive performance. BDNF: brain-derived neurotrophic factor.

In conclusion, oxidative stress, neuroinflammation, apoptosis, and mitochondrial dysfunction are involved in the pathophysiology of many diseases, including neurodegenerative disorders. The modulation of these pathways represents valuable therapeutic targets. Similarly, PPARγ agonists are able to counteract deleterious processes, positively influencing the pathology of diseases through the promotion of neuronal health and survival pathways, in particular in cases of cognitive impairment (Figure 2). Indeed, PPARγ agonists could be potential therapeutic approaches for the improvement of cognitive performance in different pathological conditions, as discussed in this review. However, future investigations should be focused on improving the safety and/or efficacy of PPARγ agonists and are necessary to better understand the underlying mechanisms by which PPARγ exerts its beneficial activities.
